# Supplementary material for: Infection with a newly designed dual fluorescent reporter HIV-1 effectively identifies latently infected CD4+ T cells
Source: eLife. 2021 Apr 9;10:e63810. doi: 10.7554/eLife.63810 (PMC8041464; doi:10.7554/eLife.63810)
Supplement: Figure 6—source data 1. [file elife-63810-fig6-data1.docx]

**Source data 1.** Characteristics of ART-suppressed, aviremic people living with HIV-1 (PLWH) in this study.

| **Participant** | **Sex** | **Treatment Regimen** | **ART**  **Duration** | **Plasma Viral Load (copies/ml)** | **CD4 Count (cells/ul)** | **CD8 Count (cells/ul)** |
| --- | --- | --- | --- | --- | --- | --- |
| Patient 1 | M | TDF+FTC+EFV | >24 months | <50 | 756 | 1695 |
| Patient 2 | M | TDF+FTC+EFV | >24 months | <50 | 650 | 550 |
| Patient 3 | M | TDF+FTC+EFV | >24 months | <50 | 696 | 830 |
| Patient 4 | M | TDF+FTC+EFV | >24 months | <50 | 497 | 432 |
| Patient 5 | M | TDF+FTC+EFV | >24 months | <50 | 471 | 453 |

TDF: tenofovir disoproxil; FTC: emtricitabine; EFV: efavirenz; M, male;
